# Evaluation of inflammation adjustment methods to assess iron deficiency using longitudinal data from norovirus human challenge trials

**DOI:** 10.1371/journal.pgph.0003964

**Published:** 2024-12-19

**Authors:** Yi-An Ko, Parminder S. Suchdev, Jiaxi Geng, Hanqi Luo, Melissa F. Young, Anne M. Williams

**Affiliations:** 1 Department of Biostatistics and Bioinformatics, Rollins School of Public Health, Emory University, Atlanta, Georgia, United States of America; 2 Department of Global Health, Rollins School of Public Health, Emory University, Atlanta, Georgia, United States of America; 3 Department of Pediatrics, Emory School of Medicine, Atlanta, Georgia, United States of America; University of Cape Town Faculty of Health Sciences, SOUTH AFRICA

## Abstract

Accounting for inflammation is necessary to assess iron deficiency using ferritin. A limitation of existing inflammation-adjustment methods is reliance on cross-sectional data to evaluate method performance. The study objective was to evaluate three inflammation-adjustment methods using longitudinal data from two controlled trials where apparently healthy adults (n = 52) were exposed to norovirus. Correction factors (CF), the Biomarkers Reflecting Inflammation and Nutritional Determinants of Anemia (BRINDA) regression correction (BRC), and restricted cubic splines (RCS) were used to adjust the influence of inflammation on ferritin using alpha-1-acid glycoprotein (AGP) and/or C-reactive protein (CRP). Blood was collected at baseline (day 0, pre-exposure to norovirus) and at 9 time points post-exposure (days 1, 2, 3, 4, 7, 14, 21, 28, and 35). Inflammation-adjusted ferritin concentrations were compared with 1) baseline, 2) endline, 3) the average of baseline and endline, and 4) predicted ferritin concentrations among subjects with infection, expressed as percent difference. The predicted ferritin concentrations were modeled using data from 26 subjects without infection in a linear mixed model. Adjusting for CRP or AGP, the median differences between adjusted ferritin using CF, BRC, and RCS were respectively [0.2%, 2.5%], [-22.2%, -20.8%], [-16.7%, -7.1%] compared with the average of baseline and endline values and were 0%, [-16.8%, -18.5%], [-8.9%, -2.8%] compared with predicted ferritin concentrations. For BRC, adjusting for both CRP and AGP tended to result in more over-adjustment of ferritin compared to using a single inflammatory protein. The BRC appeared to overcorrect ferritin in this study setting, while the CF yielded adjusted ferritin concentrations closer to the average baseline and endline concentrations and the predicted concentrations. Longitudinal studies with larger sample sizes exposed to other infectious agents are needed to further evaluate inflammation-adjustment methods and the need for including multiple inflammation biomarkers.

## Introduction

Some micronutrient biomarkers are affected by the acute-phase response [1–3]. Notably, ferritin is a positive acute-phase protein [4], and without adjusting for inflammation the prevalence of iron deficiency (defined by serum ferritin concentrations) can be underestimated by as much as 25% [5, 6]. The acute-phase response is an innate, immune-mediated inflammatory process accompanied by elevation of certain acute-phase proteins, such as C-reactive protein (CRP) or α-1-acid glycoprotein (AGP) in response to infection, inflammation, injury, or trauma. The World Health Organization (WHO) released updated guidance in 2020 that recognizes the complexity of measuring iron status using ferritin in areas of “widespread infection or inflammation” [7].

Various methods to adjust serum ferritin for inflammation have been used and are recommended by WHO [7]. One method is to increase cut-off values for ferritin concentrations to as high as 70μg/L to define iron deficiency. Another method is to exclude individuals with inflammation when estimating the prevalence of iron deficiency. Although both methods may seem straightforward to implement, they do not take into account the dynamic relationships between the continuous biomarkers of iron status and inflammation (i.e., varying degrees of increase in biomarker based on inflammation status) and likely result in biased estimates of deficiency [7, 8]. Applying arithmetic correction factors and utilizing regression models, including quantile regression, better account for the dynamic relationship between ferritin and inflammation [9–11]. Evidence has consistently demonstrated that adjusting ferritin for inflammation increases the prevalence of iron deficiency compared with unadjusted values [5, 6, 9, 12, 13].

Although it is now common practice to account for inflammation when assessing iron status using ferritin, consensus is lacking on the most accurate method. Comparisons of inflammation adjustment methods are hindered because they are often limited to cross-sectional data [5, 6, 11, 12, 14–17]. As a result of one single measurement per subject in cross-sectional studies, the actual micronutrient status (“ground truth”) is not available among individuals undergoing inflammation. To the best of our knowledge, there has been only one related study that used a longitudinal study setting. Wessells et al. (2019) utilized blood collected at two time points among asymptomatic Burkinabé children and compared inflammation-adjusted micronutrient concentrations within individuals whose inflammation status changed during the observation period [18]. To further evaluate inflammation adjustment methods for ferritin, we used data collected from norovirus challenge trials that included data pre-exposure and after resolution of inflammatory response [19, 20]. The time-varying fluctuations of ferritin concentrations and inflammatory proteins from this unique dataset of up to 10 data points per individual provided the opportunities to examine the accuracy of different inflammation adjustment methods.

Our objective was to examine two commonly used methods to adjust for inflammation as well as a data-driven approach. Specifically, we evaluated correction factors (CF) [5, 9], the Biomarkers Reflecting Inflammation and Nutritional Determinants of Anemia (BRINDA) regression correction (BRC) [6, 10], and restricted cubic splines (RCS) regression correction to adjust serum ferritin for inflammation using single inflammation proteins: CRP, APG, or in combination (CRP and AGP).

## Methods

The data came from apparently healthy adults who participated in norovirus challenge trials (NCT00313404 and NCT00674336) in hospital settings in the United States between 2006 and 2009 [19, 20]. A total of 64 participants were inoculated by ingesting either groundwater or oysters seeded artificially with norovirus. Venous blood samples were collected at 10 time points: baseline (day 0) and post-exposure days (1, 2, 3, 4, 7, 14, 21, 28 and 35). Emory University Institutional Review Board approved both trials. Among them, 26 became infected as defined by a norovirus-positive stool or vomitus sample tested (by real-time PCR) during follow-up. The current study included 52 subjects, namely, the 26 infected and 26 uninfected (no norovirus detected), who were matched for age. Among the 26 infected participants, 19 (73%) were symptomatic. The details of sample selection and laboratory methods were described previously [21]. Data were accessed for the current research purposes on March 11, 2021. The authors of this study had no access to information that could identify individual participants. The study was a secondary data analysis and was considered exempt by the Institutional Review Board at Emory University.

Descriptive statistics were used to summarize baseline characteristics for study subjects stratified by infection status. The following methods were applied to adjust ferritin for both CRP and AGP, CRP alone, and AGP alone: 1) CF proposed by Thurnham et al. [5, 9], 2) BRC [6, 10], and 3) adjustment using a RCS model. To mimic regression correction in a population survey with variable inflammation burden, the 10^th^ percentiles of CRP (0.18 mg/L) and AGP (0.38 g/L) based on all the baseline data points (day 0) from 52 participants regardless of infection status were used to define low inflammation for the BRC approach [6]. Using splines has the potential advantage of capturing the true shape of the relationship between ferritin and inflammatory proteins. For RCS models, ln(ferritin) was regressed on ln(CRP) and/or ln(AGP) with 3 knots placed at the 10th, 50th, and 90th percentiles.

For each individual, adjusted ferritin concentrations using each method at each time point were compared with 1) baseline (measured on day 0 before norovirus exposure), 2) endline, 3) average of baseline and endline, and 4) predicted ferritin concentrations. The endline ferritin concentration was calculated by taking the average of ferritin measured on day 14 and later, because not everyone completed the entire follow-up, and ferritin concentration remained relatively constant after the first 2 weeks. The overall ferritin levels were found to decrease over time among subjects without infection likely because of iron depletion from repeated blood draws (**Table 1**) [11]. To account for this decline in ferritin over time, adjusted ferritin was compared with the average of baseline and endline ferritin concentrations. We further compared the adjusted with the predicted ferritin. To calculate the predicted ferritin, we assumed that the trajectory of ferritin measurements among subjects without infection represented the decline of ferritin over time in this study. A linear mixed model was used by regressing ln(ferritin) on time point (i.e., day, treated as a categorical variable) with subject-specific random intercepts using data from subjects without infection. We calculated the predicted value of ferritin for each time point (as the predicted ferritin) for those with infection using subject-specific intercept and the fixed effect estimates of time points, assuming they shared the same trajectory over time. Subsequently, adjusted ferritin was compared with the derived, predicted ferritin for those with infection and compared with unadjusted ferritin for those without infection. In addition, adjusted ferritin was compared with unadjusted ferritin across all time points for subjects without infection and to baseline values (prior to norovirus exposure) for all subjects.

**Table 1:** Characteristics of norovirus challenge subjects, stratified by resultant infection from norovirus exposure (n = 52).

	Uninfected (N = 26)	Infected (N = 26)	Overall (N = 52)
Age (years)	26.7 (7.9)	26.7 (7.5)	26.7 (7.6)
Sex (female)	14 (54%)	16 (62%)	30 (58%)
**Iron deficiency at baseline**	5 (19%)	3 (12%)	8 (16%)
Ferritin (μg/L)			
Day 0 (baseline)	58.3 [6.4, 166.0]	56.6 [7.7, 160.0]	56.9 [6.4, 166.0]
Day 3	62.6 [4.6, 168.0]	112 [11.1, 180.0]	83.2 [4.6, 180.0]
Day 4	59.8 [4.7, 171.0]	90.8 [10.1, 183.0]	71.2 [4.7, 183.0]
Day 7	53.6 [8.1, 167.0]	53.1 [12.0, 155.0]	53.6 [8.1, 167.0]
Day 14	42.6 [5.8, 163.0]	42.3 [8.2, 172.0]	42.3 [5.8, 172.0]
Day 28	37.1 [5.7, 159.0]	31.1 [6.4, 167.0]	35.9 [5.7, 167.0]
CRP (mg/L)			
Day 0 (baseline)	1.04 [0.03, 10.90]	0.79 [0.10, 10.90]	0.98 [0.03, 10.90]
Day 3	0.63 [0.01, 10.70]	16.0 [0.14, 77.90]	3.76 [0.01, 77.90]
Day 4	0.60 [0.06, 9.78]	9.27 [0.50, 50.30]	2.91 [0.06, 50.30]
Day 7	1.23 [0.10, 15.90]	1.41 [0.31, 11.70]	1.30 [0.10, 15.90]
Day 14	1.12 [0.06, 17.30]	0.74 [0.12, 11.10]	0.88 [0.06, 17.30]
Day 28	0.71 [0.03, 13.10]	0.75 [0.10, 18.20]	0.75 [0.03, 18.20]
AGP (g/L)			
Day 0 (baseline)	0.64 [0.27, 1.15]	0.56 [0.31, 0.98]	0.59 [0.27, 1.15]
Day 3	0.60 [0.29, 1.11]	0.89 [0.33, 2.26]	0.71 [0.29, 2.26]
Day 4	0.54 [0.29, 1.27]	0.94 [0.48, 1.61]	0.76 [0.29, 1.61]
Day 7	0.65 [0.27, 1.20]	0.72 [0.40, 1.18]	0.67 [0.27, 1.20]
Day 14	0.61 [0.37, 1.16]	0.56 [0.29, 1.28]	0.60 [0.29, 1.28]
Day 28	0.61 [0.38, 1.08]	0.51 [0.18, 1.38]	0.57 [0.18, 1.38]

Mean (standard deviation), frequency count (percentage), and median [minimum, maximum] are reported. Iron deficiency is defined as serum ferritin <15 μg/L at baseline (day 0 prior to norovirus exposure). CRP = C-reactive protein AGP = α-1-acid glycoprotein.

To evaluate each inflammation-adjustment method, we calculated the percent difference in ferritin concentration (using the original scale for ease of interpretation) for each observation using [adjusted ferritin–compared value)/compared value] × 100%. The results were summarized using descriptive statistics and the distributions were visualized using boxplots to compare across methods. We further compared the proportions of data points classified as iron deficient, as defined by a serum ferritin level of <15 μg/L according to WHO guidelines, across different methods of adjusting for inflammation. Additionally, we conducted a sensitivity analysis by comparing iron deficiency rates using a recently identified serum ferritin cutoff of 25 μg/L [22–24].

## Results

Of the 52 subjects (30 females) with mean age 27 years (range: 19–51 years), 26 (50%) were infected after being exposed to norovirus. The majority (N = 44, 85%) had their last observations on day 35, 6 (12%) had the last observations on day 28, and 1 on day 7 and 1 on day 4. One subject was missing baseline measurement but had complete data until day 35. Ferritin concentrations and inflammatory proteins stratified by infection status are summarized in **Table 1**, and **S1 Fig** shows their trajectories over time. Median (min, max) CRP concentrations (mg/L) were 1.0 (0, 10.9), 3.8 (0, 77.9), 2.9 (0, 50.3) and 1.3 (0.1, 15.9) on days 0, 3, 4, and 7, respectively. Median (min, max) AGP concentrations (g/L) were 0.6 (0.3, 1.2), 0.7 (0.3, 2.3), 0.8 (0.3, 1.6) and 0.7 (0.3, 1.2) on days 0, 3, 4, and 7, respectively. After being exposed to norovirus, of the 219 measurements in subjects without infection, 194 (89%), 14 (6%), 1 (0.4%), and 10 (5%) were in reference (AGP ≤ 1 g/L and CRP ≤ 5 mg/L), incubation (AGP ≤ 1 g/L and CRP > 5 mg/L), early convalescence (AGP > 1 g/L and CRP > 5 mg/L), and late convalescence (AGP > 1 g/L and CRP ≤ 5 mg/L), respectively. Of the 226 measurements among those with norovirus infection, 161 (71%), 40 (18%), 18 (8%), and 7 (3%) were in reference, incubation, early convalescence, and late convalescence, respectively.

**Fig 1:**
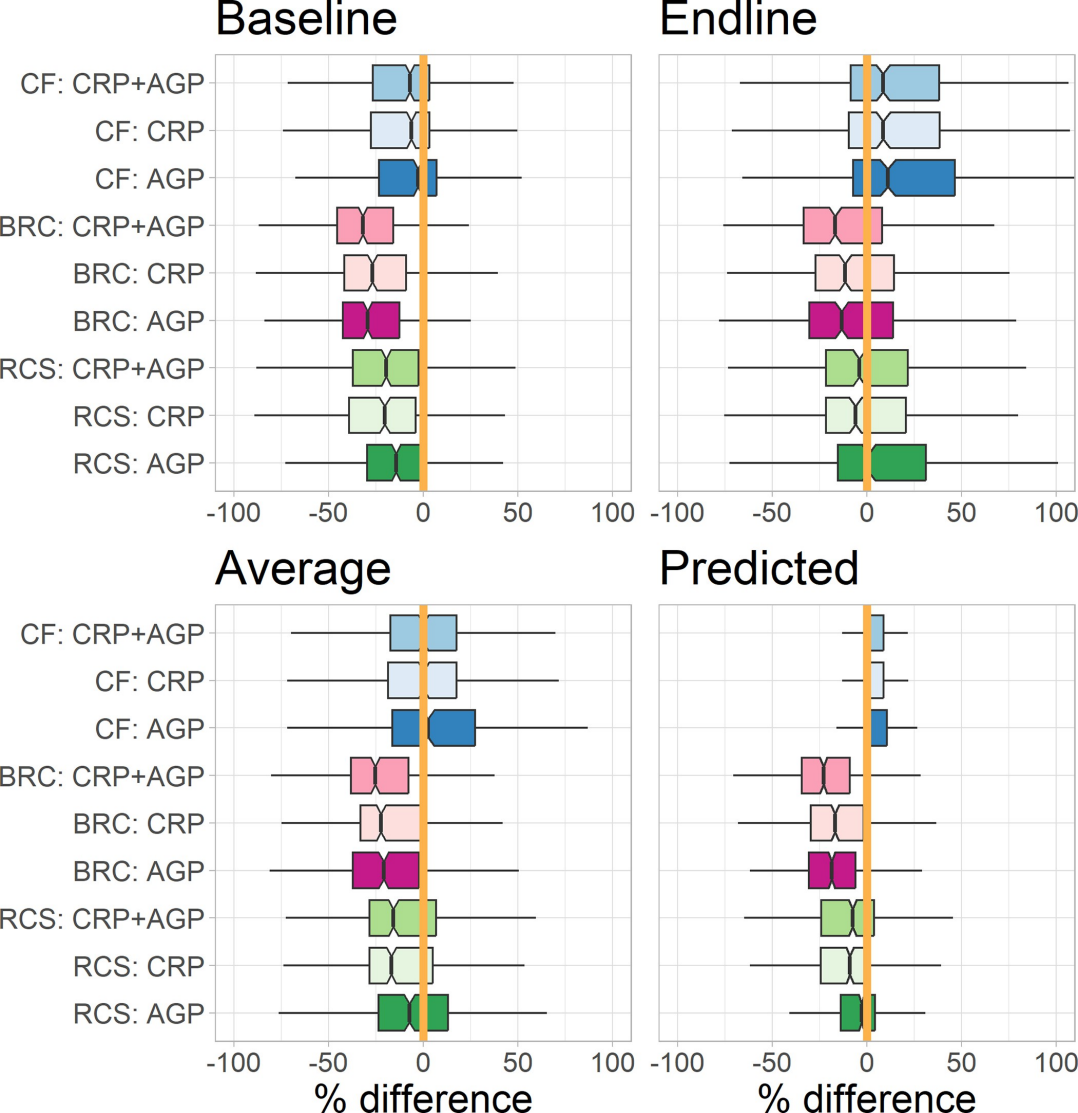
Distributions of percent difference in ferritin comparing inflammation-adjusted values with the baseline, endline, average of baseline and endline, and predicted (for those with infection) and unadjusted (for those without infection) values among adults exposed to norovirus, using Correction Factors (CF), BRINDA Regression Correction (BRC), and restricted cubic spines (RCS). Inflammatory biomarkers were α-1-acid glycoprotein (AGP) and C-reactive protein (CRP). The midline indicates the median difference in percentage, with the upper and lower limits of the box being the third and first quartile (75th and 25th percentile) respectively. The whiskers extend up to 1.5 times the interquartile range from the top (bottom) of the box to the furthest data within that distance. The reference values (or deciles) used for CRP and AGP were 0.18 mg/L and 0.38 g/L.

**Table 2** and **Fig 1** summarizes the comparison of adjustment methods for ferritin (CF, BRC, RCS, utilizing single or combined inflammatory proteins) with baseline, endline, average of baseline and endline, and predicted ferritin concentrations. The CF method adjusting for CRP and/or AGP and RCS adjusting for AGP yielded a median difference (i.e., median value of differences between the adjusted and comparison values for all data points) closet to 0% among the three methods. When compared with the average of baseline and endline ferritin concentrations, the median differences using CF were 0.1–2.5% adjusting for CRP and/or AGP. Using RCS adjusting for AGP, the median difference was -7.1%. When compared with predicted ferritins, the median differences were 0% for CF and -2.8% for RCS with AGP adjustment (**Table 2**). Notably, using CF, the results adjusting for CRP alone were comparable to those adjusting for both CRP and AGP based on the distributions. BRC generally underestimated ferritin concentration by 17–25% (median differences) when compared with the average or predicted ferritin concentrations.

**Table 2:** Inflammation-adjusted ferritin concentration was compared with baseline, endline, average of baseline and endline ferritin, and predicted (including unadjusted ferritin for those uninfected) ferritin for each individual at each time point.

Method	Baseline	Endline	Average	Predicted
CF: CRP + AGP	-7.0 (-26.6, 3.4)	8.7 (-8.4, 38.2)	0.1 (-17.3, 17.7)	0.0 (0.0, 8.8)
CF: CRP	-6.3 (-27.6, 3.4)	8.7 (-9.5, 38.5)	0.2 (-18.6, 17.7)	0.0 (0.0, 8.8)
CF: AGP	-2.8 (-23.3, 7.1)	11.0 (-7.1, 46.4)	2.5 (-16.2, 27.4)	0.0 (0.0, 10.7)
BRC: CRP + AGP	-31.9 (-45.4, -15.8)	-16.7 (-33.3, 8.2)	-25.3 (-38.1, -7.7)	-22.9 (-34.3, -9.1)
BRC: CRP	-26.8 (-41.6, -8.9)	-11.4 (-27.1, 14.5)	-22.2 (-33.0, -0.9)	-16.8 (-29.6, -1.6)
BRC: AGP	-29.2 (-42.3, -12.5)	-13.4 (-30.2, 13.8)	-20.8 (-37.1, -2.1)	-18.5 (-30.5, -5.9)
RCS: CRP + AGP	-19.5 (-37.0, -2.5)	-4.1 (-21.5, 21.7)	-15.7 (-28.3, 6.8)	-7.4 (-24.1, 3.9)
RCS: CRP	-20.3 (-39.1, -4.1)	-5.9 (-21.4, 20.7)	-16.7 (-28.3, 5.1)	-8.9 (-24.2, 1.4)
RCS: AGP	-14.3 (-29.5, -0.1)	1.1 (-15.3, 31.2)	-7.1 (-23.4, 13.0)	-2.8 (-13.8, 4.2)

Data presented are Median (Lower Quartile, Upper Quartile) of percent differences in ferritin concentrations using correction factors (CF), BRINDA regression correction (BRC), restricted cubic spines (RCS) adjusting for c-reactive protein (CRP) and/or α-1-acid glycoprotein (AGP).

In addition, adjusted ferritin was compared with unadjusted ferritin across all time points for subjects without infection as well as with baseline values (prior to norovirus exposure) for those who were infected. Since the majority (89–95%) of person-day ferritin observations were not adjusted using CF, this approach produced a median difference of 0% [0%, 0%]. BRC underestimated ferritin concentrations by median differences of -26% [-35%, -16%], -21% [-30%, -11%], and -24% [-33%, -13%] when adjusting for both CRP and AGP, CRP only, and AGP only. RCS also slightly underestimated ferritin concentrations by median differences of -9.1% [-21.2%, -2.9%], -11.5% [-22.3%, -4.6%], and -5.2% [-14.8%, -0.9%] when adjusting for both CRP and AGP, CRP only, and AGP only.

**Fig 2:**
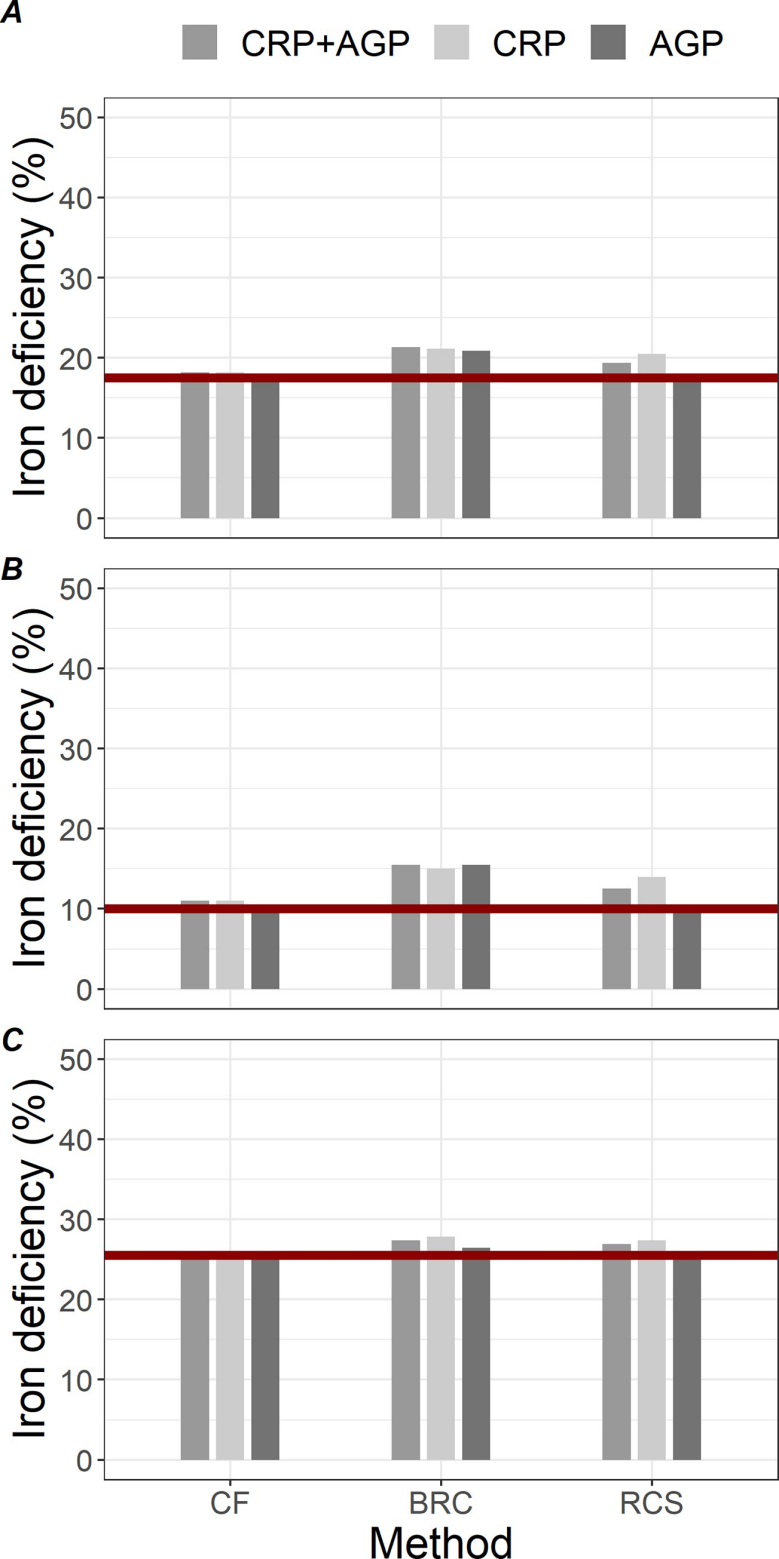
Percentage of data points categorized as iron deficient (serum ferritin <15 μg/L) based on inflammation-adjusted ferritin concentration for (A) all observations (n = 445 time-person observations), (B) among subjects with infection excluding baseline measurements (n = 200), and (C) among those without infection including baseline measurements (n = 219). Inflammation adjustment methods are Correction Factors (CF), BRINDA Regression Correction (BRC), and restricted cubic spines (RCS). Each method was adjusted for α-1-acid glycoprotein (AGP) and/or C-reactive protein (CRP). The horizontal line indicates the percentage of iron deficiency using raw, unadjusted ferritin concentrations.

**Fig 2** illustrates the percentages of data points categorized as iron deficient based on inflammation-adjusted ferritin concentrations for all observations (**Fig 2A**), among subjects with infection excluding baseline (pre-exposure) measurements (**Fig 2B**), and among those without infection including baseline measurements (**Fig 2C**). Comparing with approximately 20% iron deficiency using unadjusted ferritin concentrations, CF demonstrated similar percentages of iron deficiency across different scenarios, while using BRC consistently resulted in slightly higher percentages of iron deficiency particularly among those with infection. RCS adjusted for AGP alone produced comparable percentage of iron deficiency to CF. **S2 Fig** shows results using serum ferritin cutoff of 25 μg/L.

## Discussion

We evaluated three inflammation-adjustment methods using three combinations of inflammatory proteins, for a total of nine approaches compared to 4 reference concentrations of ferritin: baseline, endline, the average of baseline and endline, and the predicted ferritin concentration, using serial measurements from a small sample of adults exposed to norovirus. This evaluation illustrated that the CF method with both CRP and AGP, or with CRP only, yielded inflammation-adjusted ferritin most similar to the comparison groups, specifically the average (of baseline and endline ferritins) or predicted ferritin concentration. However, the CF did not adjust most ferritin measurements because incubation, early and late convalescence were uncommon based on the cutoffs of CRP > 5 mg/L or AGP > 1g/L. The RCS method using only AGP also appeared to be a reliable method for this setting, suggesting that flexible, data-driven slopes, may be superior to a single coefficient for each inflammatory protein as the BRC uses. The BRC method yielded a median difference in ferritin that ranged from -17% to -25% when compared with average (baseline and endline) or predicted values. Considering both CRP and AGP in the adjustment seemed to yield the farthest difference from 0, compared to using only CRP or only AGP in the adjustment method. Specifically, CF with CRP, RCS with AGP, and BRC with AGP or CRP appeared to provide sufficient adjustment as opposed to adjustment of both inflammatory biomarkers under various scenarios.

In this study, CF may have provided results most similar to the comparison groups since the adjustment thresholds are much higher than the other two methods. In contrast, BRC, which underestimated ferritin concentrations, and therefore overestimated iron deficiency, may not be well-suited for a generally healthy adult population exposed to an infectious agent that does not generate a large acute phase inflammatory response. While using BRC, we identified two participants whose data were considered as potential influential points. We performed a sensitivity analysis by excluding the two participants’ data. Regardless, there were no appreciable differences in the results. Compared to BRC (linear regression), RCS can model non-linear relationships, but they also carry an inherent risk of overfitting and are sensitive to data scarcity. We used a relatively small number of knots and quantiles for default knot locations [25]. Alternatively, Akaike’s Information Criterion (AIC) or cross-validate can be implemented to select a knot number. **S3 Fig** shows the RCS models with scattergrams. Due to the high variability in within-subject ferritin concentrations observed in our small study, further investigation and development of inflammation adjustment methods may be necessary before applying them to individual cases in clinical practice.

It is necessary to adjust for inflammation carefully when estimating micronutrient concentrations and deficiencies, but there is no consensus on method to adjust ferritin for inflammation. Studies evaluating inflammation adjustment of micronutrient biomarkers have predominately used cross-sectional data [5, 6, 11, 12, 14–17]. Previously, Wessells et al. [18] investigated the association between within-individual changes in CRP and AGP and micronutrient biomarkers longitudinally in children, and they confirmed that micronutrient concentrations, including ferritin, changed with infection and inflammation status by the same direction and similar magnitudes. They further compared CF and BRC adjustment methods using cross-sectional (by ignoring within-subject correlation) and longitudinal models and found significant differences in micronutrient concentrations. Regardless, the estimated prevalence of micronutrient deficiencies was similar. This is one of the very few studies that compared inflammation-adjustment methods in a longitudinal study setting. More recently, Urbanski et al. used cohort studies to establish a ferritin / CRP ratio as an alternative biomarker for iron deficiency in the presence of inflammation or comorbidities [26]. Inflammation adjustment for micronutrient concentrations is still an active research area in population and clinical research.

The global burden of iron deficiency is concentrated among women of reproductive age and younger children. Thus, most studies assessing iron status focus on these population groups. In our prior meta-analysis that included multiple nationally representative surveys of iron and inflammation status among women of reproductive age, the 10^th^ percentile of CRP and AGP used to define low inflammation for the BRC were 0.16 mg/L and 0.53 g/L, respectively [10]. In contrast, the secondary analysis of two clinical trials presented here includes apparently healthy young adults and 42% were men. The 10^th^ percentiles of CRP and AGP that we used to define low inflammation to apply the BRC method were 0.18 mg/L and 0.38 g/L, respectively. The reference value for AGP was much lower than that for women of reproductive age, a more micronutrient-deficient population typical of national micronutrient surveys, suggesting more ferritin observations would be adjusted for inflammation via BRC in this study when using data-driven deciles. In contrast, only 29% of the data points (and even less when adjusting for CRP or AGP alone) were adjusted using CF in our study. This may have contributed to similar iron deficiency proportions using the CF method vs. using unadjusted ferritin concentrations among subjects with and without infection.

The study’s strength is having repeated measures of iron and inflammation status collected before and after exposure to norovirus using standardized procedures, where half of those exposed became infected with norovirus. Another strength of this study was the number of comparisons that were done to account for the decline in iron status over the course of the study, ostensibly from repeated blood draws. Rather than only comparing to baseline (pre-infection) and endline (post-exposure and when the acute phase response effect was no longer apparent) ferritin concentrations, we also considered the average of those values and modeled the predicted ferritin concentrations among those that were infected based on the ferritin concentration patterns over time among the uninfected. We chose to make qualitative comparisons based on descriptive statistics rather than to do formal statistical testing because similarity (or equivalence) was the focus and the clinically acceptable difference in ferritin concentrations has not been established. The study’s constraints include having a moderate sample size and a limited scope of generalizability, since apparently healthy adults were the only possible demographic to enroll into a human norovirus challenge trial.

## Conclusion

We provide invaluable insight of how different ferritin-adjustment methods perform in a small norovirus-exposed trial, which can serve as a foundation for future larger studies that may include other micronutrients and variable inflammatory responses in different study populations. This evaluation can serve as a reference to inform which ferritin inflammation-adjustment method would be the most appropriate as investigators are challenged by the multitude of options. Finally, we hope that this study can provide evidence to improve refinement of inflammation-adjustment methods to assess iron deficiency prevalence more easily and accurately.

## Supporting information

S1 Fig Individual trajectories of natural logarithm transformed serum ferritin (μg/L), α-1-acid glycoprotein (AGP, g/L), and C-reactive protein (CRP, mg/L) at baseline (day 0) and days after exposed to norovirus. Locally weighted scatterplot smoothing lines and the corresponding 95% confidence bands were added to illustrate the overall trends for infected and uninfected groups.
(DOCX)

S2 Fig Percentage of data points categorized as iron deficient (serum ferritin <25 μg/L) based on inflammation-adjusted ferritin concentration for (A) all observations (n = 445 time-person observations), (B) among subjects with infection excluding baseline measurements (n = 200), and (C) among those without infection including baseline measurements (n = 219). Inflammation adjustment methods are Correction Factors (CF), BRINDA Regression Correction (BRC), and restricted cubic spines (RCS). Each method was adjusted for α-1-acid glycoprotein (AGP) and/or C-reactive protein (CRP). The horizontal line indicates the percentage of iron deficiency using raw, unadjusted ferritin concentrations.
(DOCX)

S3 Fig Relationships between ferritin and AGP (log-transformed) using restricted cubic spline models along with scattergrams. (DOCX)
