# Supplementary material for: Evaluation of inflammation adjustment methods to assess iron deficiency using longitudinal data from norovirus human challenge trials
Source: PLOS Glob Public Health. 2024 Dec 19;4(12):e0003964. doi: 10.1371/journal.pgph.0003964 (PMC11658468; doi:10.1371/journal.pgph.0003964)
Supplement: S1 Fig — Locally weighted scatterplot smoothing lines and the corresponding 95% confidence bands were added to illustrate the overall trends for infected and uninfected groups. (DOCX) [file pgph.0003964.s001.docx]

**
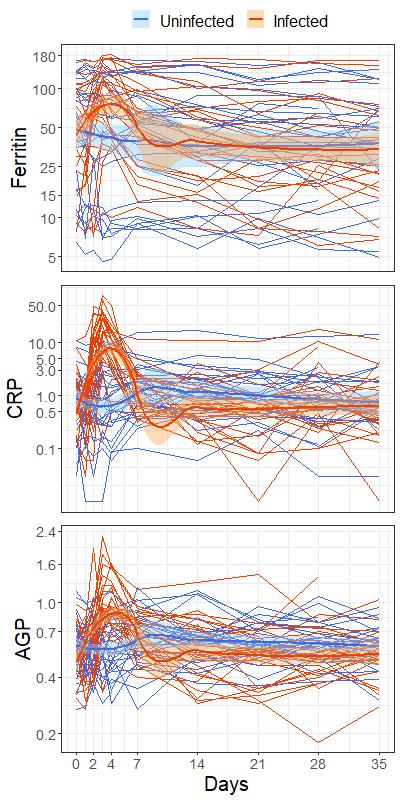
**

**S1 Fig:** Individual trajectories of natural logarithm transformed serum ferritin (µg/L), α-1-acid glycoprotein (AGP, g/L), and C-reactive protein (CRP, mg/L) at baseline (day 0) and days after exposed to norovirus. Locally weighted scatterplot smoothing lines and the corresponding 95% confidence bands were added to illustrate the overall trends for infected and uninfected groups.
