# Supplementary material for: Evaluation of inflammation adjustment methods to assess iron deficiency using longitudinal data from norovirus human challenge trials
Source: PLOS Glob Public Health. 2024 Dec 19;4(12):e0003964. doi: 10.1371/journal.pgph.0003964 (PMC11658468; doi:10.1371/journal.pgph.0003964)
Supplement: S2 Fig — Percentage of data points categorized as iron deficient (serum ferritin <25 μg/L) based on inflammation-adjusted ferritin concentration for (A) all observations (n = 445 time-person observations), (B) among subjects with infection excluding baseline measurements (n = 200), and (C) among those without infection including baseline measurements (n = 219). Inflammation adjustment methods are Correction Factors (CF), BRINDA Regression Correction (BRC), and restricted cubic spines (RCS). Each method was adjusted for α-1-acid glycoprotein (AGP) and/or C-reactive protein (CRP). The horizontal line indicates the percentage of iron deficiency using raw, unadjusted ferritin concentrations. (DOCX) [file pgph.0003964.s002.docx]

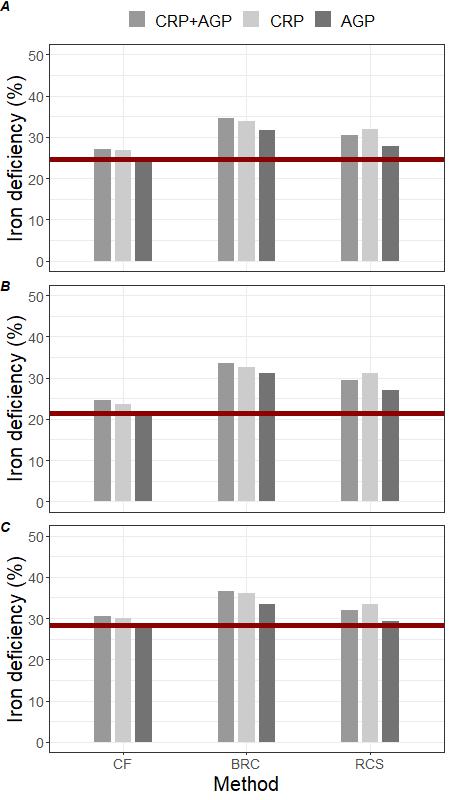


**S2 Fig:** Percentage of data points categorized as iron deficient (serum ferritin <25 µg/L) based on inflammation-adjusted ferritin concentration for (A) all observations (n=445 time-person observations), (B) among subjects with infection excluding baseline measurements (n=200), and (C) among those without infection including baseline measurements (n=219). Inflammation adjustment methods are Correction Factors (CF), BRINDA Regression Correction (BRC), and restricted cubic spines (RCS). Each method was adjusted for α-1-acid glycoprotein (AGP) and/or C-reactive protein (CRP). The horizontal line indicates the percentage of iron deficiency using raw, unadjusted ferritin concentrations.
