# Supplementary material for: Evaluation of inflammation adjustment methods to assess iron deficiency using longitudinal data from norovirus human challenge trials
Source: PLOS Glob Public Health. 2024 Dec 19;4(12):e0003964. doi: 10.1371/journal.pgph.0003964 (PMC11658468; doi:10.1371/journal.pgph.0003964)
Supplement: S3 Fig — (DOCX) [file pgph.0003964.s003.docx]

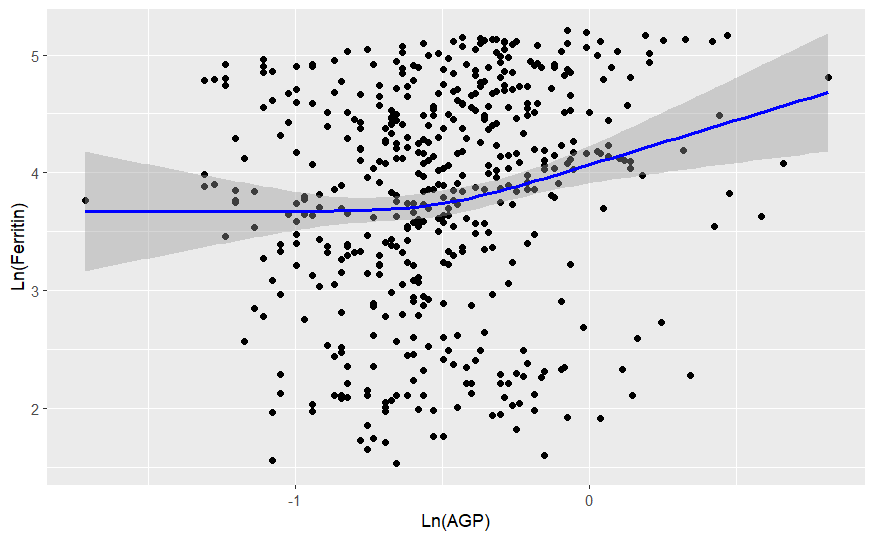

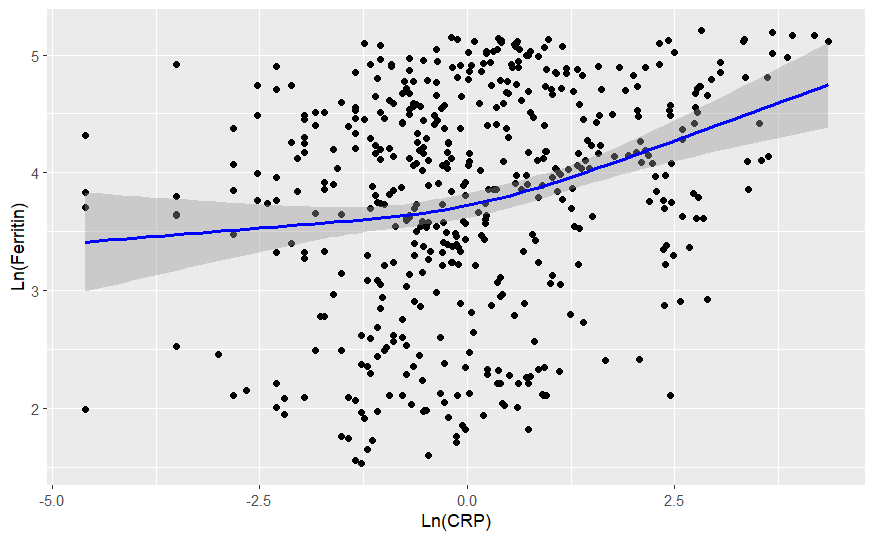


**S3 Fig:** Relationships between ferritin and AGP (log-transformed) using restricted cubic spline models along with scattergrams.
